# Exploring practice and perspectives on shared decision-making about osteoporosis medicines in Fracture Liaison Services: the iFraP development qualitative study

**DOI:** 10.1007/s11657-024-01410-6

**Published:** 2024-06-19

**Authors:** Laurna Bullock, Fay Manning, Ashley Hawarden, Jane Fleming, Sarah Leyland, Emma M. Clark, Simon Thomas, Christopher Gidlow, Cynthia P. Iglesias-Urrutia, Joanne Protheroe, Janet Lefroy, Sarah Ryan, Terence W. O’Neill, Christian Mallen, Clare Jinks, Zoe Paskins

**Affiliations:** 1 Centre for Musculoskeletal Health Research, School of Medicine, Keele University, Staffordshire, UK; 2 University of Exeter Medical School, Devon, UK; 3 Haywood Academic Rheumatology Centre, Midlands Partnership University NHS Foundation Trust, Stoke-On-Trent, Staffordshire UK; 4 Cambridge Public Health, University of Cambridge, Cambridge, UK; 5 Addenbrooke’s Hospital Fracture Liaison Service, Cambridge University Hospitals NHS Trust, Cambridge, UK; 6 Royal Osteoporosis Society, Bath, UK; 7 Bristol Medical School, Faculty of Health Sciences, University of Bristol, Bristol, UK; 8 Prescribing Decision Support Ltd and School of Pharmacy and Bioengineering, Keele University, Staffordshire, UK; 9 Centre for Health and Development, Staffordshire University, Stoke-On-Trent, Staffordshire, UK; 10 Department of Health Sciences, University of York, York, UK; 11 Danish Centre for Healthcare Improvement (CHI), Aalborg University, Aalborg, Denmark; 12 School of Nursing and Midwifery, Keele University, Staffordshire, UK; 13 Centre for Epidemiology Versus Arthritis, University of Manchester, Manchester, UK; 14 NIHR Manchester Biomedical Research Centre, Manchester University NHS Foundation Trust, Manchester Academic Health Sciences Centre, Manchester, UK

**Keywords:** Osteoporosis, Fracture Liaison Services, Qualitative, Intervention development, Consultation, iFraP, Shared decision-making

## Abstract

**Summary:**

Interviews and focus groups with patients, FLS clinicians, and GPs identified challenges relating to clinical and shared decision-making about bone health and osteoporosis medicines. Findings will inform the development of the multicomponent iFraP intervention to address identified training needs and barriers to implementation to facilitate SDM about osteoporosis medicines.

**Purpose:**

The iFraP (improving uptake of Fracture Prevention treatments) study aimed to develop a multicomponent intervention, including an osteoporosis decision support tool (DST), to support shared decision-making (SDM) about osteoporosis medicines. To inform iFraP intervention development, this qualitative study explored current practice in relation to communication about bone health and osteoporosis medicines, anticipated barriers to, and facilitators of, an osteoporosis DST, and perceived training needs.

**Methods:**

Patients attending an FLS consultation (*n* = 8), FLS clinicians (*n* = 9), and general practitioners (GPs; *n* = 7) were purposively sampled to participate in a focus group and/or telephone interview. Data were transcribed, inductively coded, and then mapped to the Theoretical Domains Framework (TDF) as a deductive framework to systematically identify possible barriers to, and facilitators of, implementing a DST.

**Results:**

Inductive codes were deductively mapped to 12 TDF domains. FLS clinicians were perceived to have specialist expertise (knowledge). However, clinicians described aspects of clinical decision-making and risk communication as difficult (cognitive skills). Patients reflected on decisional uncertainty about medicines (decision processes). Discussions about current practice and the proposed DST indicated opportunities to facilitate SDM, if identified training needs are met. Potential individual and system-level barriers to implementation were identified, such as differences in FLS configuration and a move to remote consulting (environmental context and resources).

**Conclusions:**

Understanding of current practice revealed unmet training needs, indicating that using a DST in isolation would be unlikely to produce a sustained shift to SDM. Findings will shape iFraP intervention development to address unmet needs.

**Supplementary Information:**

The online version contains supplementary material available at 10.1007/s11657-024-01410-6.

## Introduction

Shared decision-making (SDM) is described by the National Institute for Health and Care Excellence (NICE) as a joint process that involves the patient and healthcare professional working together to make decisions based on evidence and on the person’s individual preferences, beliefs, and values [1]. Despite the international agenda to improve SDM [2] and national osteoporosis guidance recommending the provision of information as a core component of management [3], people with osteoporosis report dissatisfaction with the information they receive. A UK population survey of 1188 people with osteoporosis and fragility fractures identified ‘improving access to information from health professionals’ as the number one patient priority for research [4]. Insufficient or inaccessible patient information that does not address health literacy needs limits patient involvement in the consultation and drug treatment decisions [5–7].

In UK practice, many discussions about osteoporosis medicines take place in the context of specialist ‘Fracture Liaison Services’ (FLSs, or sometimes referred to as Fracture ‘Prevention’ Services). These are typically nurse-led services that seek to systematically investigate and assess people aged 50 and over with osteoporotic type fragility fractures to identify if osteoporosis medicine is appropriate and make other recommendations to reduce falls and fracture risk. In the UK, these services usually make a recommendation to the patients’ general practitioner (GP) or primary care practitioner to prescribe a drug treatment. Uptake of osteoporosis medicine is poor and reportedly poorer than other long-term conditions with adherence estimated at 16–60% at 1 year for those that choose to start taking an osteoporosis medicine [8]. Evidence from national audits of specialist FLSs suggests that only 23% of patients who are recommended medicine are recorded as taking it 1 year later [9]. A failure of clinicians to adequately explain medicine benefits and harms has been blamed, at least in part, for poor treatment uptake [10].

NICE’s SDM guidelines recommend that, where available, clinicians should use decision aids or decision support tools (DSTs) to support SDM [1]. DSTs are designed to help people to be involved in decision-making about healthcare options, supporting people to make informed, value-based decisions. Evidence suggests that DSTs increase decisional certainty, knowledge, and accuracy of risk perception [11] and can address concerns about safety and/or doubts about need for medicine, thereby increasing patient commitment to medicines. Despite this evidence, existing osteoporosis DSTs fail to comprehensively meet international quality standards and patient needs [12] and are not widely used in UK clinical practice.

The iFraP (improving uptake of Fracture Prevention drug treatment) study aims to develop and evaluate a theoretically informed complex intervention, including an osteoporosis DST to be used on the computer during FLS appointments, to support SDM, with a long-term aim of improving patient commitment to osteoporosis medicines [13]. This paper forms part of the multimethod project to develop the prototype iFraP intervention, alongside three additional studies, including (1) an evidence synthesis of existing osteoporosis DSTs to examine quality and effectiveness [12]; (2) a review of patient information about osteoporosis to evaluate quality and optimal language for talking about osteoporosis [5]; and (3) a Delphi survey with patients, carers, and clinicians to gain consensus on intervention content, informed by an evidence review of relevant clinical guidelines and behavioural theories [14]. Further details of the iFraP intervention development work are described elsewhere [13]. This qualitative study aimed to explore current practice in relation to communication about osteoporosis medicine, anticipated barriers to, and facilitators of, using a computerised osteoporosis DST, and perceived training needs.

## Methods

This qualitative focus group study is underpinned by the pragmatist paradigm, utilising the best methods to investigate real‐world problems, allowing for the use of multiple sources of data and knowledge to answer research questions that are contextually relevant [15]. This paradigm aligns with the iFraP multimethod intervention development study using the ‘best methods’ to answer unanswered questions about context and defining and understanding the problem for intervention [13].

### Stakeholder engagement

Our community of practice (CoP) met frequently throughout the iFraP study [13] to bring together people with expertise who share a common concern or interest [16]. Expert stakeholders included FLS clinicians, people with osteoporosis and/or lived experience of osteoporosis medicines, pharmacists, GPs, osteoporosis specialists, representatives from the Royal Osteoporosis Society (ROS) and Health Literacy UK, and a behaviour change expert with expertise in medicine adherence. The CoP supported the development of topic guides and handouts (described in detail later) that facilitated data generation and interpretation of findings, including any outstanding uncertainties and implications for iFraP intervention development.

Public involvement was integral to iFraP development to ensure the final intervention was targeted to patient priorities. Two dedicated patient advisory group (PAG) meetings were convened to discuss this focus group study. The first face-to-face meeting facilitated development of topic guides to ensure that content was relevant and understandable. The second PAG meeting facilitated data interpretation and was conducted using video conferencing software. Public involvement has been reported with reference to the Guidance for Reporting Involvement of Patients and the Public (GRIPP) 2 criteria [17].

### Participants and recruitment

Patients attending an FLS consultation, who were recommended drug treatment, were provided with information packs during their consultation by two FLS sites in the UK West Midlands region. Patients who returned an expression of interest indicating interest in participating were purposively selected to represent maximum variation in gender and age.

We also recruited nurses and allied health professionals who consulted with patients in UK FLSs (described as ‘FLS clinicians’) and GPs who work in the catchment area of a UK FLS, with experience of consulting with patients who have been seen in their local FLS. Focus groups were advertised by varied national and regional clinical networks (e.g. the ROS Allied Health Professional networks (including FLS Champions), Midlands Bone Interest Group, and the North Staffordshire GP federation). Snowball sampling via existing clinical contacts of the study team and stakeholders was also utilised. Clinicians who indicated interest in participating were purposively selected to represent maximum variation in FLS location.

### Data collection

We aimed to conduct two focus groups with patients, two with FLS clinicians, and one with GPs, with approximately 4–8 participants in each group. Four focus groups were facilitated by an experienced qualitative researcher (LB (female), with assistance from FM (female) or AH (male)) between November 2019 and March 2020. Focus group facilitators debriefed immediately following data collection. The remaining patient focus group was cancelled due to the COVID-19 pandemic. Patients scheduled to take part in the cancelled focus group were offered participation in individual telephone interviews. The interviewer (LB) met with other study team members on a regular basis to discuss interview data collection. Data collection was completed by June 2020. All participants provided informed consent.

Topic guides guided discussions to explore current practice and to identify potential barriers to and facilitators of DST use in FLS, informed by evidence gathering, CoP/PAG views, and the Theoretical Domains Framework (TDF) [18] (see Additional file 1). The refined TDF includes 14 domains to examine determinants of current and desired behaviours that may influence intervention implementation, including knowledge; skills (interpersonal, cognitive, and physical skills); social/professional role and identity; beliefs about capabilities; optimism; beliefs about consequences; reinforcement; intentions; goals; memory, attention, and decision processes; environmental context and resources; social influences; emotion; and behavioural regulation.

Participants were provided with a series of handouts (sent via post for telephone interviews, Additional file 2) containing images of an example osteoporosis DST, including a series of headers such as ‘benefits’, ‘common side effects’, and ‘rare side effects’ to facilitate discussion. It was explained to participants that each header, if on a computer screen, could be selected by the clinician, to reveal more information about that topic area. Participants were also shown examples of icon arrays of 100 people, often used in DSTs, presenting an illustrative risk of fracture in the next 10 years, with and without an osteoporosis medicine. This handout provided opportunity to explore participant perceptions of visual presentations of risk, absolute (rather than relative) risk, and natural frequencies (for example, 10 in 100), as recommended by NICE to facilitate SDM [1].

Ethical permission for the study was given by North West-Greater Manchester West Research Ethics Committee (reference number: 19/NW/0559).

### Analysis

Data collection and analysis were undertaken concurrently so that topic guides could be modified iteratively. We followed the seven stages of framework analysis [19], using the TDF as a deductive framework.

Focus groups and interviews were recorded, transcribed verbatim (by an external transcription company), and anonymised (stage 1). Field notes were made immediately after each focus group and interview, followed by extensive familiarisation with the data by revisiting recordings and transcripts (stage 2).

The first few anonymised transcripts were open coded independently by two researchers with experience in qualitative data collection and analysis (LB/ZP) (stage 3). Discussions about coding facilitated development of the working analytical coding framework (stage 4). The analytical coding framework was then systematically applied across participant datasets (step 5). Codes with similar meanings were grouped together to form broader categories and charted to a framework matrix (step 6).

To facilitate data interpretation about barriers and enablers (stage 7), we then deductively mapped the analytical coding framework to the 14 domains of the refined TDF[18]. Open coding followed by deductive mapping to an existing theoretical framework is a commonly used analytical process to ensure that context and detail is maintained [20], as recommended by the framework method [19]. The TDF was preselected as the deductive framework to facilitate insight into barriers and facilitators to intervention implementation [21]. This mapping exercise was completed by LB, with detailed discussions with two other experienced qualitative researchers (ZP and CJ). This process involved examining codes and data extracts to explore how they fit (or did not fit) within the parameters of each theoretical domain. A further iteration of the framework matrix was produced using Microsoft Excel to exemplify data extracts from each participant group (patient, FLS clinician, and GP), relating to each analytical code and TDF domain (see Table Additional File 3 for an excerpt of the framework matrix, including mapped TDF domain). Organising the qualitative findings using the TDF domains is a commonly adopted approach in applied qualitative intervention development research to systematically identify relevant barriers to behaviour change [21, 22].

## Results

Four focus groups were conducted: one with patients (*n* = 4), two with FLS clinicians (*n* = 4 and *n* = 5), and one with GPs (*n* = 7), lasting between 1 h 20 min and 2 h 25 min. Of the five patient participants who had agreed to participate in a second focus group (before this had to be cancelled due to COVID19 restrictions), four consented (with one declining participation due to changing life circumstances) and completed individual telephone interviews, lasting between 1 h 18 min and 1 h 40 min. Demographic characteristics of participants are detailed in Table 1.

**Table 1:** Participant demographics

Patient participants (*n* = 8)	
Gender *n* (%)	
Female	6 (75)
Male	2 (25)
Age mean (range)	
	68.8 (60–76)
Patient self-reported osteoporosis medicine recommendation *n* (%)	
Yes	6 (75)
Unknown	2 (25)
Data collection method	
Focus group	4 (50)
Telephone interview	4 (50)
Fracture Liaison Service clinicians (*n* = 9)	
Gender *n* (%)	
Female	9 (100)
Professional role	
Nurse	8 (88)
Allied health professional	1 (12)
General practitioners (*n* = 7)	
Gender *n* (%)	
Female	4 (57)
Male	3 (43)
Years in practice mean (range)	
	15 (1–30)

The coding framework included 25 codes, which were subsequently mapped to 12 of the 14 TDF domains (see Additional file 3). No codes were mapped to TDF domain optimism and behavioural regulation. Findings are presented below, with exemplary quotes, split into each deductive TDF domain. Under each domain, findings first relate to current FLS practice, followed by participant perspectives of an osteoporosis DST.

### Knowledge

FLS clinicians reflected upon their expertise in bone health, perceiving themselves as having good knowledge and understanding of osteoporosis and associated medicines. In contrast, GPs described their perceived lack of knowledge of osteoporosis and reliance on the FLS clinician expertise.

‘I do wonder whether I know enough to explain it back to the patient’ (GP03)

Despite recently attending their FLS appointment, patients’ understanding of osteoporosis and associated medicines varied. Some patients had gaps in their understanding following their FLS appointment.

‘I’ve heard about osteoporosis but I didn’t know exactly what osteoarthritis was. I don’t know the difference.’ (P03)

### Interpersonal and cognitive skills

FLS clinicians reflected that they often had the skills to make a clinical judgement, to decide whether to recommend osteoporosis medicine and, if so, which medicine to recommend. This was reiterated by GPs who spoke positively of FLS recommendations. FLS clinicians reportedly tailored explanations, by using simple language, to take into account patient characteristics that may hinder their understanding (e.g. patient education level, language barriers, presence of cognitive impairment).

‘You try and make it as basic as possible for some individuals and just keep the information that you give them as low key and as easy as possible.’ (FLS08)

Despite acknowledging factors that may impede patient understanding, some FLS clinicians reportedly did not check patient understanding or personalise information to the patient’s values, needs, or concerns.

‘I recognise that it’s something that we could do but currently we don’t. I don’t think we find out what their values are before or what their expectations are.’ (FLS01)

Describing the potential consequences of osteoporosis and the benefits and risks of osteoporosis medicine in a balanced way was perceived an important skill of FLS clinicians. However, FLS clinicians at times faced challenges when accurately interpreting and communicating benefit and risk to patients in an understandable way. When FLS clinicians were shown the absolute risk reduction (the recommended method of communicating risk), some FLS clinicians thought that this minimised the ‘true’ effectiveness of the medicine. Incorrect interpretation and communication of risk inflated the perceived benefits of osteoporosis medicine, with some quoting that osteoporosis medicines ‘reduce the risk of fracture by 50%’ when the absolute risk reduction may be less than this.

‘I think in some ways because it goes against the figures that you’re given for taking treatments a little bit. Most of them say it will reduce their risk of having a fracture by 50% but that’s just showing 10%.’ (FLS09)

### Physical skills

FLS clinicians suggested that they had the IT skills to use the computer during their current practice (e.g. to calculate FRAX® scores [23]). These IT skills were viewed as sufficient to use a computerised DST, but some recognised the need for training to implement a DST into their current practice workflow.

‘It’s about actually developing a consultation skill that would involve the use of a tool like this. It’s going to change how we do an assessment or a consultation’ (FLS09)

### Memory, attention, and decision processes

FLS clinicians had a multitude of factors to consider when deciding whether to recommended osteoporosis medicine to a patient (such as bone density scan results, patient age, potential medicine contraindications).

‘We all come up with a different point of view as to what is actually the best way to manage this individual. Sometimes, like you say, it’s a really, really grey area, isn’t it?’ (FLS08)

FLS clinicians reported that, in some circumstances, they were unsure whether they should recommend osteoporosis medicine, with many wanting to discuss the decision with others, e.g. their consultant colleagues.

‘We normally just ask our consultants for their opinion before we will consider a recommendation’ (FLS06)

Similarly, patients faced challenges when deciding whether they wanted to accept the recommended medicine. One patient, despite being recommended an osteoporosis medicine, did not feel sufficiently informed to make a decision. This same patient perceived the DST as having potential to address their information needs about osteoporosis medicine and facilitate decision-making.

‘I may be taking it now as a result of being able to discuss this (…) This here [referring to example DST], it lets you see what the benefits would be. I still don’t know what the benefits are of taking them.’ (P03)

Unpreparedness for the information received during the appointment was reported by patients, with many not expecting to receive a diagnosis of osteoporosis, making it more difficult to consider options and make decisions.

‘I didn’t know what to expect really, so I was being guided by her. I didn’t know that if you had bone fragility that you’d have to take something really big for it.’ (P03)

Patients’ perceptions of the amount of information received during the FLS consultation varied. Some indicated that they received too much information leading to cognitive overload, whereas others reflected that they needed more information to support their involvement in the decision-making process, highlighting the need for tailored information giving. FLS clinicians, GPs, and patients each suggested that a DST with embedded visuals (pictures and videos) may support patient memory and attention and in turn minimise cognitive load.

‘I think to have something visual like this would help to split the discussion up into groups so you are going to be able to remember it better’ (P05)

A printable summary of the consultation was also recommended by clinicians to support evidence-based and consistent conversations outside of the FLS consultation.

‘It might be useful if we then get a summary of what’s been discussed [in FLS] (…) when we have the conversation then, we can say, “We can see that you’ve discussed *X*, *Y* and *Z*.” It’s clear what’s been discussed already’ (GP06)

### Social influences

Patients’ preconceptions were important to understand and address. FLS clinicians, GPs, and patients commented upon the potential influences of family, friends, and neighbours that shape patients’ perceptions of osteoporosis and osteoporosis medicines.

‘You also get quite a few patients that have already come with preconceived ideas because their friend or neighbour has already been on that medication.’ (FLS01)

### Environmental context and resources

When deciding which osteoporosis medicine to recommend to the patient, in the absence of contraindications, FLS clinicians and GPs recommended the first-line option, with local prescribing protocols and medicine costs frequently cited as the justification. This external influence impeded patient involvement in decisions about medicine ‘options’ and preferences.

‘We got this directive from pharmacy and NHS England that we’ve got to prescribe the cheapest one. There is not a lot of discussion really.’ (GP03)

Some FLS clinicians questioned how a DST would be implemented in different FLS models of care that varied in the amount of face-to-face patient-clinician contact. It was identified that some FLSs did not conduct the assessment and provide a medicine recommendation on the same day, whilst others did not recommend medicine at all.

‘Every service is different, isn’t it?’ (FLS06)

FLS clinicians also raised concerns associated with the resources necessary to implement the iFraP intervention, including the IT infrastructure to support a computerised DST, time-limited consultation, and resources needed to distribute iFraP information resources.

‘I think it [DST] would make the assessment a lot longer’ (FLS03)

### Emotion

FLS clinicians thought it necessary to heighten the importance and relevance of osteoporosis, whilst taking care to minimise patient fears that may impede willingness to start taking medicines.

‘I think that percentage of fracture risk is really powerful for the patient to start treatment and to take it. If you’re saying, “You’ve got a 15% chance of fracturing your hip. Whereas your risk of side effects from the medication is lower,” that’s powerful.’ (FLS09)

### Professional role, identity, and reinforcement

The roles of the patient, FLS clinician, and GP were distinct. Patients chose whether they did or did not want to accept the osteoporosis medicine which was often decided on and recommended by the FLS clinician. Patients were not, however, involved in the choice of osteoporosis medicine.

‘Sorry, no, they don’t have a choice [laughter]. (FLS01)

‘We say, “this is what we’d recommend your GP prescribes for you. That’s the treatment that would be recommended for you.”’ (FLS02)

Conversely, one FLS clinician commented that it was not part of their role to discuss medicine, a task more appropriate for a GP.

‘The thing is the GP is the best placed person to know that patient in terms of what other medications they’re on and what they can and can’t take.’ (FLS05)

The GPs’ role was to consider the FLS recommendations in the context of the patient’s wider circumstances (such as their living situation, mobility, co-morbidities, and medicines) and to support long-term management of osteoporosis.

‘When the patient comes back to us, we put it more into context of what else is going on in their lives and see if it fits.’ (GP07)

### Beliefs about capabilities

The knowledge and skills held by FLS clinicians contributed to participant’s perceived capability to deliver current practice.

Although most participants thought that patients would be able to engage with a computerised DST, some expressed concern for people with eyesight impairment or low health and digital literacy, for example.

‘We’re all being expected to be computer literate and many people over 80 aren’t and are feeling slightly excluded.’ (P08)

### Beliefs about consequences

When envisaging use of the DST during an FLS appointment, FLS clinicians expressed concern regarding numerous potential unintended consequences. Clinicians thought that increasing patient involvement by using the DST may allow patients to direct the structure and content of the consultation, impeding the FLS clinician’s ability to form an appropriate management plan.

‘If we’re going to leave it to the patient to say what’s important to them, we may lose quite a lot of information that enables us to give them the best clinical pathway for them’ (FLS08)

Some FLS clinicians also suggested that increased use of the computer might reduce the person-centred nature of the consultation.

‘I think you’d also tend to focus on the computer than the patient.’ (FLS09)

Some FLS clinicians and patients suggested that using natural frequencies and icon arrays to communicate the absolute risk reduction of future fracture when taking osteoporosis medicines may create perceptions that the medicine was not ‘worthwhile’ in the context of their perceived risk of side effects. This was deemed to have potential negative consequences for rates of osteoporosis medicine uptake.

‘Only ten people are going to avoid breaking a bone and we’re starting them on this medication with all these side effects. If it was me, I would say, “Is it worth me taking it?”’ (FLS08)

### Intentions

Despite describing potential negative consequences of the risk presentations on medicine uptake, clinicians also suggested that icon arrays may positively facilitate patient involvement in the decision-making process and support clinicians to communicate the effectiveness of osteoporosis medicine in an understandable way, an aspect of current FLS consultations that clinicians described as challenging.

‘I think it would generally help because people understand something pictorial much more than just figures.’ (FLS09)

Conversely, FLS clinicians who perceived themselves as ‘experts’ thought that the iFraP DST would add limited value to their consultation, with the value of the DST being for clinicians without specialist expertise in osteoporosis, or lower grade staff.

‘I don’t think it’s any different to what we already do’ (FLS02)

### Goals

FLS clinicians perceived the goal of FLSs to support osteoporosis medicine uptake and commitment, in line with service key performance indicators. Some clinicians expressed confidence that they currently do well in achieving this goal. This goal appeared to be at odds with the goal of iFraP to increase SDM, as some FLS clinicians perceived that giving patients the autonomy to refuse medicines was a threat to adherence.

‘If they’ve got quite low bone density, to me, I want them on treatment.’ (FLS03)

## Discussion

### Summary of main findings

Person-centred care and SDM about osteoporosis medicines (decision processes) in current FLS practice was influenced by the clinician’s skills and beliefs about capabilities. Patients’ expectations for their FLS appointment were incongruent with their experiences, with many not expecting to potentially receive a diagnosis of osteoporosis or to discuss medicine options, limiting their ability to engage with decision-making discussions. FLS clinicians reflected that they regularly tailored explanations according to their perception of each patient’s level of understanding but not to the patient’s values and preferences, limiting SDM. Furthermore, communicating benefits and risks in an understandable way was difficult for FLS clinicians and GPs. Clinical decision-making is an important component of SDM; however, FLS clinicians, at times, described facing uncertainty when deciding whether to recommend osteoporosis medicines, suggesting difficulty with interpreting clinical guidelines. These findings together indicate that using a DST in isolation would be unlikely to produce a sustained shift to SDM, because of identified training needs.

Anticipated barriers and facilitators centred on the clinician’s physical skills to use, and the FLS environmental context and resources to implement, a computerised DST in FLS. FLS clinicians reported having the skills necessary to use a DST however expressed concern that use during the consultation would be detrimental to the patient-clinician relationship and would not fit within a time-limited FLS consultation. System-level (environmental) barriers were also identified. FLS clinicians and GPs were concerned that a DST would present different osteoporosis medicines to the patient as equal ‘choices’ when in fact, the first-line medicine was directed by clinical guidelines, thereby limiting choice. Providing patients with a ‘choice’ about medicines appeared to sit at odds with the FLS goal to encourage medicine adherence. Variation in FLS models of care was identified as an additional system-level contextual factor requiring consideration, given that some FLS clinicians revealed they did not discuss medicines with patients.

### Discussion with wider literature

Providing patients with understandable and accessible information, considering varied levels of health literacy, is essential to support SDM [24]. Many patients in this study did not expect to potentially receive a diagnosis of osteoporosis or to discuss medicine options. Providing patients with consistent, repeated, and accurate information in an accessible format is imperative to support patient preparedness, decision-making, and ongoing conversations with family, friends, and health professionals [25]. Furthermore, despite clinicians’ efforts, patients reflected that, at times, they did not understand information that they received during their FLS consultation, as also found in previous work [6]. This is important, as people with low health literacy neither expect nor receive SDM [26–28], despite evidence of the benefits of SDM for those with low health literacy[7, 28–30]. Involving family, friends, and/or carers in the consultation as a ‘medical visit companion’ can facilitate information exchanges and decision-making, particularly for patients with low health literacy [31, 32]. The move to remote consulting, accelerated in response to the COVID19 pandemic, may threaten this, further widening existing health inequalities[33, 34]. Communicating benefits and risks in an understandable way was difficult for clinicians, as reflected in previous literature in which clinicians often did not discuss, or simply relayed risk scores, without discussing the implications of the risk for the patient or what they could do about it [35, 36]. This study also identified that clinicians, at times, seemed to incorrectly interpret fracture risk reduction benefits of osteoporosis medicines. Patients included in this study expressed varied needs for the amount and depth of information on risks and benefits, demonstrating the need for individualised information giving [14].

Explanations tailored to patient’s values, preferences, and circumstances are a key component of person-centred care and SDM [24]. However, patient questions regarding the need for medicines were not addressed, hindering decision-making. Perceptions of treatment need are subtly different to treatment benefits and require a conceptualisation of how the proposed treatment benefits might align with individual goals. This is important because low necessity beliefs are known to be associated with lower levels of medicine adherence [37].

FLS clinicians, at times, faced uncertainty when deciding whether to recommend osteoporosis medicines, which has been identified in other clinician groups, such as GPs [38, 39]. In contrast, some FLS clinicians revealed they did not discuss or recommend medicines with patients, rather that this was the GP’s role, demonstrating variation in FLS models of care. This is of concern because GPs clearly reflected that they did not have the knowledge to discuss these medicines in detail and relied on FLS for their expert opinion and recommendations. Current national standards for FLSs require high risk patients to be recommended medicine but do not provide detailed information about where, when, or with whom these discussions with the patient should take place [3]. We therefore suggest that FLS standards [3] should be explicit about the role of the specialist FLS in this regard.

Clinicians expressed concerns when considering implementing an osteoporosis DST in practice. A DST was viewed as potentially detrimental to the patient-clinician relationship, which is consistent with previous research indicating links between increased clinician computer-use and decreased patient satisfaction [40]. However, video recordings of consultations using a DST demonstrate that when the computer screen was visible to the patient and clinician together, positive person-centred connections were created [41]. Clinicians in this study also perceived the time taken to implement a DST to be problematic, as identified in previous studies [27], a systematic review concluded that the majority of DST studies found no significant difference in duration between consultations using a DST versus usual care [42]. Finally, FLS clinicians and GPs were also concerned that a DST that guided clinical decision-making would present different osteoporosis medicines to the patient as equal ‘choices’ when in fact, the first-line medicine was directed by clinical guidelines. In previous qualitative research exploring patient perceptions of a variety of DSTs, including DSTs relating to osteoporosis, patients expressed concern that clinicians using a DST would not be directed by the patient’s medicine ‘choice’ [43].

### Implications for iFraP development

This qualitative study identified barriers and training needs to be addressed in the iFraP intervention development. A separate paper will describe in-depth how each of the intervention development studies came together to inform iFraP intervention development, including how the TDF enabled the identification and integration of evidence-based behaviour change techniques, guided by the COM-B model of behaviour [44].

DSTs used during the consultation have been described as requiring ‘minimal training for use’ [45]. However, to successfully implement SDM, it is essential to increase clinician understanding of what it entails and the potential benefits to overcome the barriers presented in this qualitative study. Providing clinicians with a DST in isolation may not be sufficient to support implementation and long-standing changes to patient-clinician interaction [46, 47]. These findings illustrate that additional intervention components, such as clinician training workshops, are necessary to address identified training needs. We specifically used these findings to design training content to address identified training needs in SDM, risk interpretation and communication, and health literacy. Training content also aimed to shift clinician attitudes regarding SDM [46] to align the goals of the intervention (to increase SDM) with the goals of FLS (to increase patient commitment to medicines) to facilitate implementation. The discordance between the goals was particularly enlightening and represents an important barrier to implementing SDM interventions which may have resonance in other contexts.

The qualitative findings demonstrated the need for additional iFraP intervention resources that complement the iFraP DST and clinician training workshops. Patients and clinicians expressed an unmet need for understandable and accessible resources that facilitate consistent ongoing discussions about osteoporosis with professional and social networks. The iFraP intervention resources will incorporate minimal text and plain language to attend to universal precautions for health literacy, including a personalised easy-read summary to provide to the patient and their GP after their FLS consultation.

The variation in FLS models of care identified in this study has important implications when considering the implementation of the proposed osteoporosis DST which may have reduced relevance in some services, where medicines are not discussed with patients. This finding necessitated a national survey of FLS usual care to quantify the number of services which discuss osteoporosis medicines with patients, in which the iFraP intervention would be relevant. The iFraP national survey of FLS usual care found that more than a quarter of responding UK FLSs did not explain osteoporosis medicines with the patient [48]. This is important given that GPs’ report lacking confidence, knowledge, and skills to discuss osteoporosis medicine.

## Strengths and limitations

Clinical stakeholders’ and public contributors have been invaluable to the design, conduct, and analysis of this research. In the early stages of project development, PAG members recommended that we conduct a focus group with GPs, who are an important component of the osteoporosis management care pathway. The inclusion of PAG members also facilitated data interpretation, with members highlighting the importance of being involved and being adequately informed in consultations about bone health.

Deductive use of the TDF as a framework ensured that barriers to implementation were systematically identified. In some circumstances, open codes were mapped to multiple TDF domains; for example, FLS clinicians discussed their knowledge and skills, which often influenced beliefs of capabilities. This, as reflected previously by other researchers, caused challenges such as repetition and difficulty clarifying boundaries between domains [49]. In contrast, there were two TDF domains to which none of the open codes were mapped: optimism and behavioural regulation. This study used the TDF prospectively to inform design of the iFraP intervention, and therefore, participants were yet to discuss factors that aimed to ‘manage or change’ their actions, as defined in behavioural regulation [18]. Deductive use of the TDF has been criticised; the overly structured application of domains may become restrictive, possibly overlooking potentially important findings [22]. To overcome this, both open coding and deductive mapping facilitated data analysis to ensure that the context and detail from open coding was maintained, retaining focus on individual and system-level considerations.

This study has a number of limitations. COVID-19 created challenges to recruitment and data collection. Pressures on FLSs, such as clinician redeployment and halting of face-to-face appointments, hindered recruitment. Despite focus groups being planned, the final patient focus group was replaced with telephone interviews. Although this minimised the extent to which group norms and processes were investigated [50], this change was necessary to abide by social distancing requirements and ensured the inclusion of varied patient perspectives. When COVID restrictions lifted, our attention had shifted to ensure that the qualitative findings informed iFraP intervention development in preparation for feasibility testing. Furthermore, the recruitment avenues and methods employed (e.g. hosting in-person clinician focus groups in the West Midlands) potentially limited the identification of service variation.

## Conclusion

This qualitative study provided in-depth understanding of current practice and potential facilitators of, and barriers to, implementing an osteoporosis DST in FLS. Importantly, clinicians described aspects of clinical decision-making and risk communication as difficult. Patients have outstanding decisional uncertainty about medicines, even following their FLS consultation. Findings support the need for enhanced clinician consultation skills training to complement the DST implementation, as findings indicate that the DST in isolation would be unlikely to produce a sustained shift to SDM. System-level barriers to implementation were identified, such as the ambiguity of clinical standards with regard to the roles of primary versus specialist care when recommending osteoporosis medicines. Another crucial barrier found was variable service configuration highlighting a need for a national survey of FLS usual care to establish the extent to which the iFraP intervention can be implemented across different FLS settings. These insights about current practice, barriers, and facilitators to DST implementation, and unmet training needs will directly inform the development of the iFraP intervention.

## Supplementary Information

Below is the link to the electronic supplementary material.

Supplementary file1 (DOCX 1416 KB)

## Data Availability

Not applicable.
