# Supplementary material for: Exploring practice and perspectives on shared decision-making about osteoporosis medicines in Fracture Liaison Services: the iFraP development qualitative study
Source: Arch Osteoporos. 2024 Jun 19;19(1):50. doi: 10.1007/s11657-024-01410-6 (PMC11186902; doi:10.1007/s11657-024-01410-6)
Supplement: Supplementary file 1 — Supplementary file1 (DOCX 1416 KB) [file 11657_2024_1410_MOESM1_ESM.docx]

**SUPPLEMENTARY MATERIAL**

**File name:** Additional file 1

**Title of data:** Example patient topic guide

**Details:** Topic guides used to support focus group and interview data collection

**File name:** Additional file 2

**Title of data:** Focus group and interview hand outs

**Details:** Handouts containing images of an example iFraP DST to facilitate discussion

**File name:** Additional file 3

**Title of data:** Analytical framework matrix mapped to the Theoretical Domains Framework domains, with example quotes

**Details:** Patient, FLS clinician, and GP quotes, presented alongside their inductive coding and TDF domain

**Additional file 1: Topic guides**

Patient topic guide

**iFraP-D: Improving consultations about Fracture Prevention**

**PATIENT FOCUS GROUP TOPIC GUIDE**

**Housekeeping**

- Welcome and introductions
- Fire alarm
- Informed consent /& consent to audio record
- Payment travel expenses
- Ground rules (confidentiality, talk one at a time, respect each other’s point of view)
- Collect demographics of participants {do they current take an osteoporosis medicine?}

**Stress that there are no right or wrong answers**

**Introduction**

- You’ve all been invited here because you have recently been to a Fracture Liaison Service and had your bone health assessed. We are interested to find out more about the conversations in the Fracture Liaison Service and how osteoporosis medicines are discussed.
- We would also like your thoughts, views and feelings about the development of a ‘new way’ of conducting Fracture Liaison Service appointments.

**Background questions related to current practice**

So first, we are going to explore your experiences of attending the Fracture Liaison Service appointment.

What were you **expecting** from the appointment? Did the appointment meet your expectations?

Do you think that the nurse explored what was **important to you**? How?

Did the nurse talk about the **possibility** of **increased risk of future broken bones**? If so, how? {**words/numbers/pictures**?}

- Did you understand your risk of broken bones?

Were any of you diagnosed with osteoporosis? If so, how was osteoporosis described? {words? scores? websites?}

What information were you given about osteoporosis medicines? (recommended/mentioned?)

- Did the nurse suggest there were medicine **options**? Which?
- Did you feel as though you had a **choice** of medicines? What made you feel this way?
- Would you have **liked a choice**?
- Did the nurse explain why osteoporosis medicine was important? (consequences)

What did the nurse tell you about the **benefits and side effects** of the medicines?

- How did you feel about this explanation?

Did you feel **confident** about your **decision** to take, or not to take osteoporosis medicines?

- What influenced your confidence?
- Do you still feel confident?

Did the nurse talk about **lifestyle choices**? For example: exercise, diet, smoking or drinking alcohol?

- How did you feel about these choices?
- Did you feel that lifestyle changes were an **alternative** to osteoporosis medicine or something to do alongside taking medicine?

Did you have **unanswered questions/confusion/ uncertainties?**

Since your appointment, have you **discussed the recommendations** with your GP?

- Have you discussed the recommendations with anyone else? {dentists, family, friends, helpline?}
- Did it change how you felt about medicines, why? why not?

**iFraP intervention:**

Now we will discuss a new way of conducting the Fracture Liaison Service appointment. Our aim is to **improve communication and the conversation** between the healthcare professional and the patient. We want to make sure the new appointment is focused on **what is important for the patient**.

**We are developing three things:**

1. **training** for **healthcare professionals**
2. a **computer-based tool** that can be used to **guide** the consultation (which I will show you examples of in a minute)
3. Information leaflets and websites for you and your GP to use after your Fracture Liaison Service appointment.

**Questions before the consultation:** We are thinking about ask patients to answer some questions about themselves before the appointment. For example, about their medical history

- How would you feel about this?
- What do you think would be the best way of asking these questions? (survey in waiting room/ipad/person – or letter through the post)
- How would you feel about answering questions about what you already know about osteoporosis or your views on medicines in general before the appointment

*Qualitative data collection prompt 2 – see Additional file 3*: I mentioned we are developing a computer based decision tool. The idea is the patient and the nurse would use this together on the computer, as part of the appointment. This sheet shows an example snapshot of the tool.

In this example, alendronic acid, an osteoporosis medicine is on the left hand side. Across the top of the decision tool are topic areas that the patient and the nurse might wish to talk about. If you click on an issue, the aid would give more information about that area, and information about any relevant risks.

- Does the tool **make sense** to you? What does the term decision tool or decision aid mean to you? What about conversation aid/treatment discussion aid?
- How would you **feel** about this tool being used to guide your conversation with the nurse discuss osteoporosis medicines? What are your thoughts?
- If this had been used in your appointment, how would it have changed the way you feel about osteoporosis medicines?
- Do you feel **confident** that you and the nurse could use this tool to discuss osteoporosis medicines?
- What **problems** do you think might occur when using the tool? What could **help** to overcome those problems?

Nurses can **estimate** an **individual fracture risk** that is **different** for each person. It is important that we clearly explain the idea of ‘fracture risk’ to the patient. I’m going to give 4 examples.

1. If someone told you that you had a **high risk** of breaking a bone over 10 years what would that mean to you?
2. If we told you that your risk of fracture was 25%, what does that mean to you?
3. What about a 1 in 4 risk, what would that mean for you?
4. *Qualitative data collection prompt 1 – see Additional file 3:* This is an example of what might be included in the decision tool for a person with a 25% risk, or a 1 in 4 risk. If we look at the picture of the left, each coloured spot represents a person like you. Over the next 10 years, for 100 people like you, 25 of them, shown in purple will break a bone, and 75 of them will not break a bone, shown in blue. Now if we take a look at the picture on the right, this is the difference that medicine could make. If there was 100 people like you taking medicines, 75 of them will still **not break** a bone, shown in blue. 15 people shown in purple would still break a bone, however 10 people, shown in yellow will be saved from breaking a bone. We do not know which one of these 100 coloured spots will be you.

- What do you think about this way of explaining fracture risk?
- Do you think this would help your understanding?
- How do you now feel about medicines lowering the risk of future fractures?

We also want to improve people’s understanding of osteoporosis.

- Do you feel you understand how osteoporosis could affect you in the future?
- Some patients have suggested that we should explain how osteoporosis could affect you. For example, by explaining the impact of a hip or spinal fracture (e.g. the back can become curved)
  - What do you think about this?
  - Does knowing the effects of osteoporosis motivate to take osteoporosis medicines or scare you?
  - Is it important to be positive? – How can the clinician explain it in a positive way?
- What if we showed a picture of someone with a spine affected by spinal fractures. How would you feel about this?

*Qualitative data collection prompt 4 – see Additional file 3:* This shows a screen that could be used at the end of the appointment

- How would you feel answering these questions? Why?
- Thinking back to your appointment, what would you have answered?
- How would you feel if your GP got a printout of these answers and your risk charts that we looked at earlier?

What **information** or **support** might you **want** after the appointment?

**Closing statement:** On behalf of the research team and Keele University I would like to “Thank you” for participating in the iFraP patient focus group and for taking the time to share with us your views today.

**Additional file 2: Focus group and interview hand outs**

Example Decision Support Tool (DST) content, which had been discussed with patient, public and key stakeholders, used adapted images from [Bone Health Choice Decision Aid - Site (mayoclinic.org)](https://osteoporosisdecisionaid.mayoclinic.org/), following collaborative work with the Mayo Clinic to investigate the quality and effectiveness of existing osteoporosis treatment decision aids^[[1]](#footnote-1)^.

Qualitative data collection prompt 1: Risk of fracture icon arrays


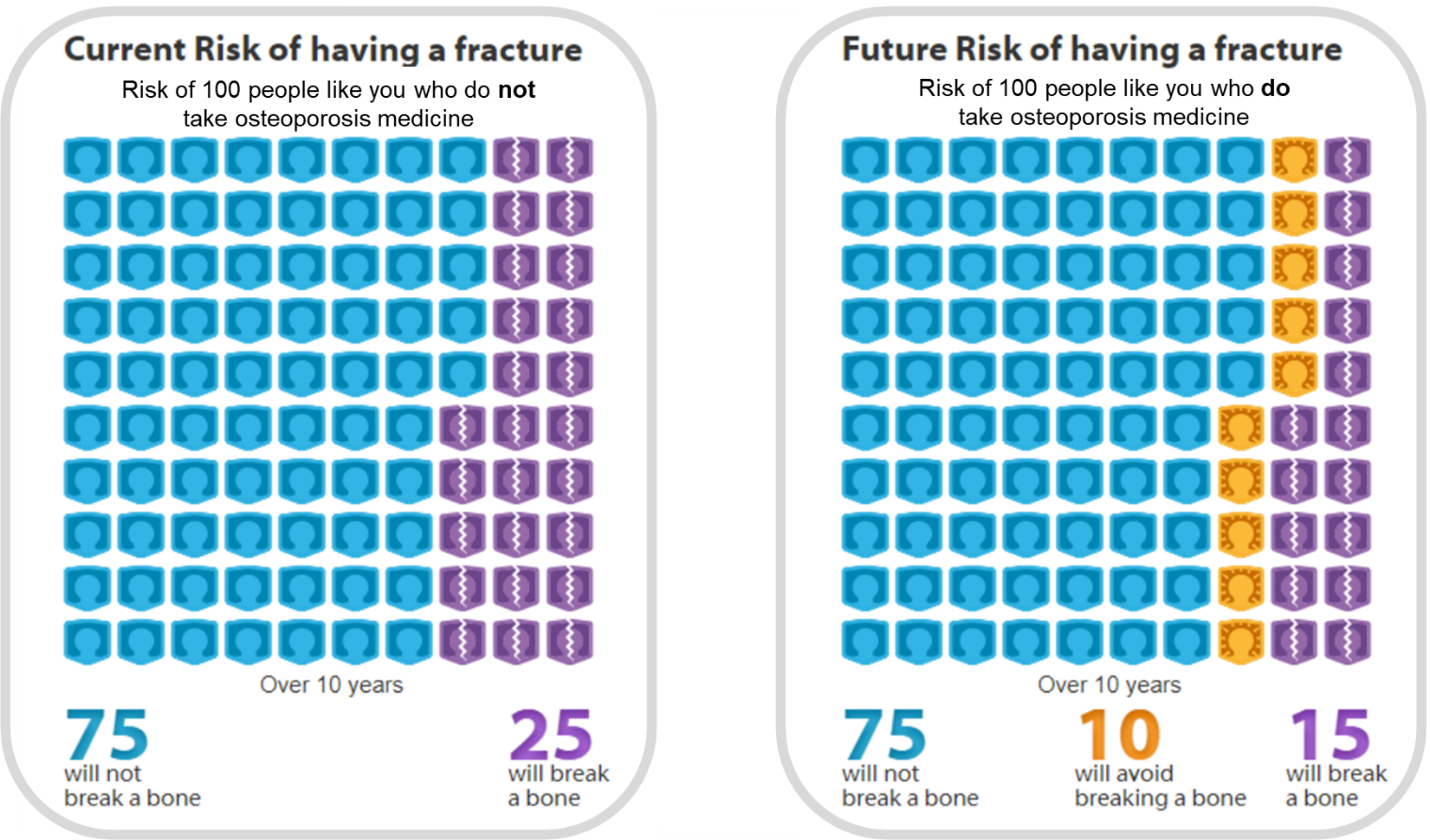


Qualitative data collection prompt 2: Example image of DST content


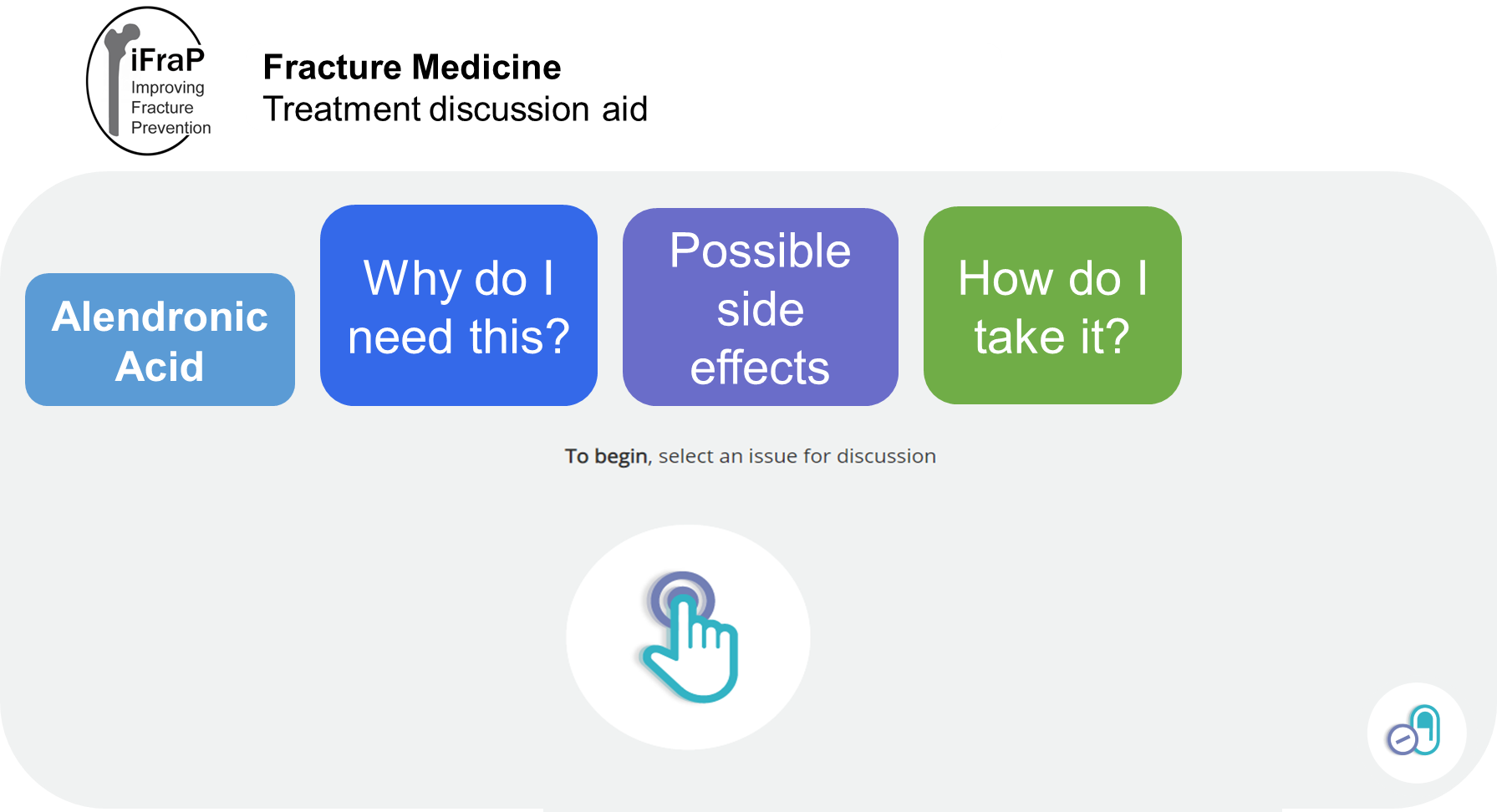


Qualitative data collection prompt 3: GP printed output (GP focus group only)


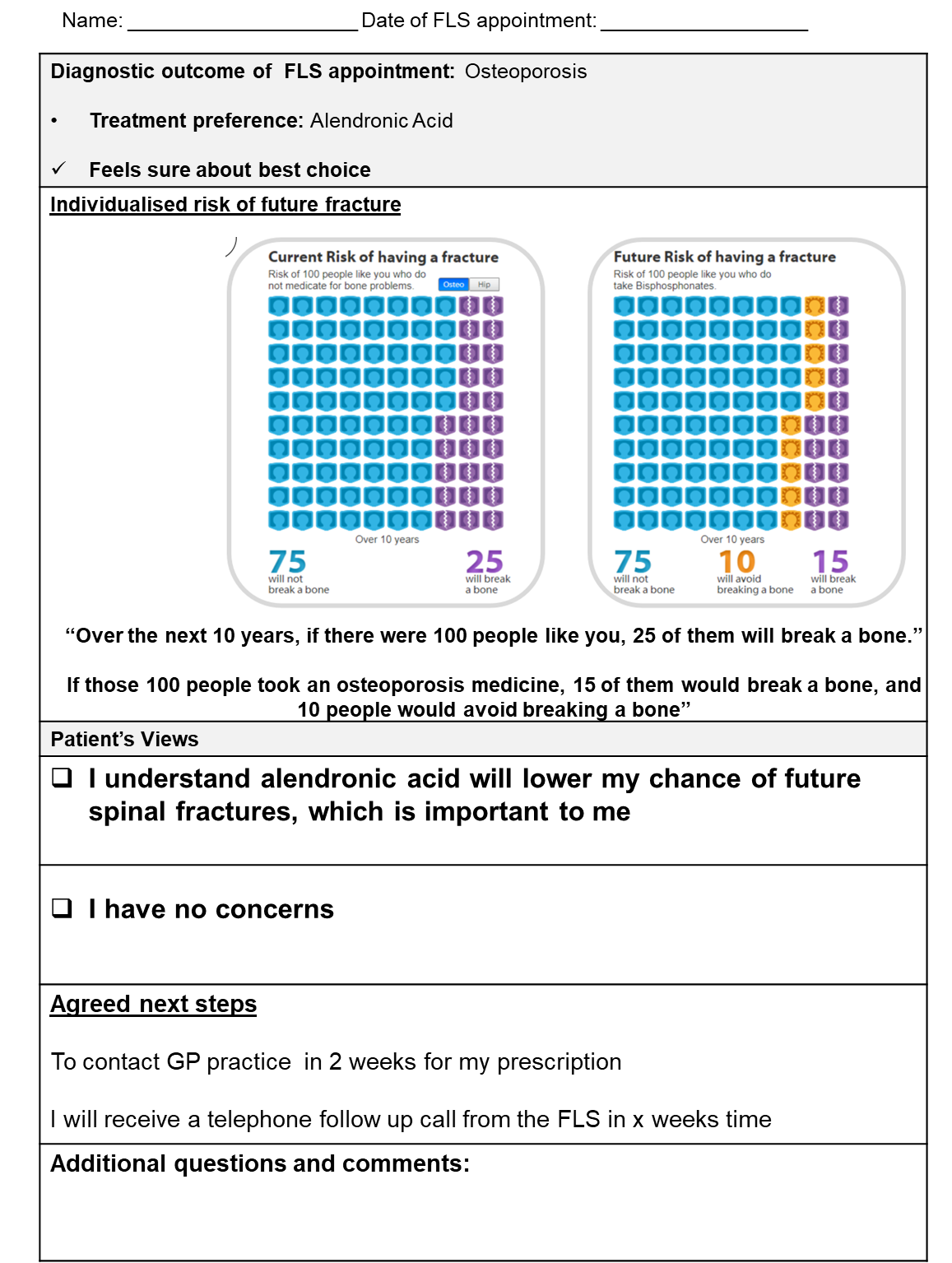


Qualitative data collection prompt 4: Necessity concerns profiling (presented at the end of the DST)


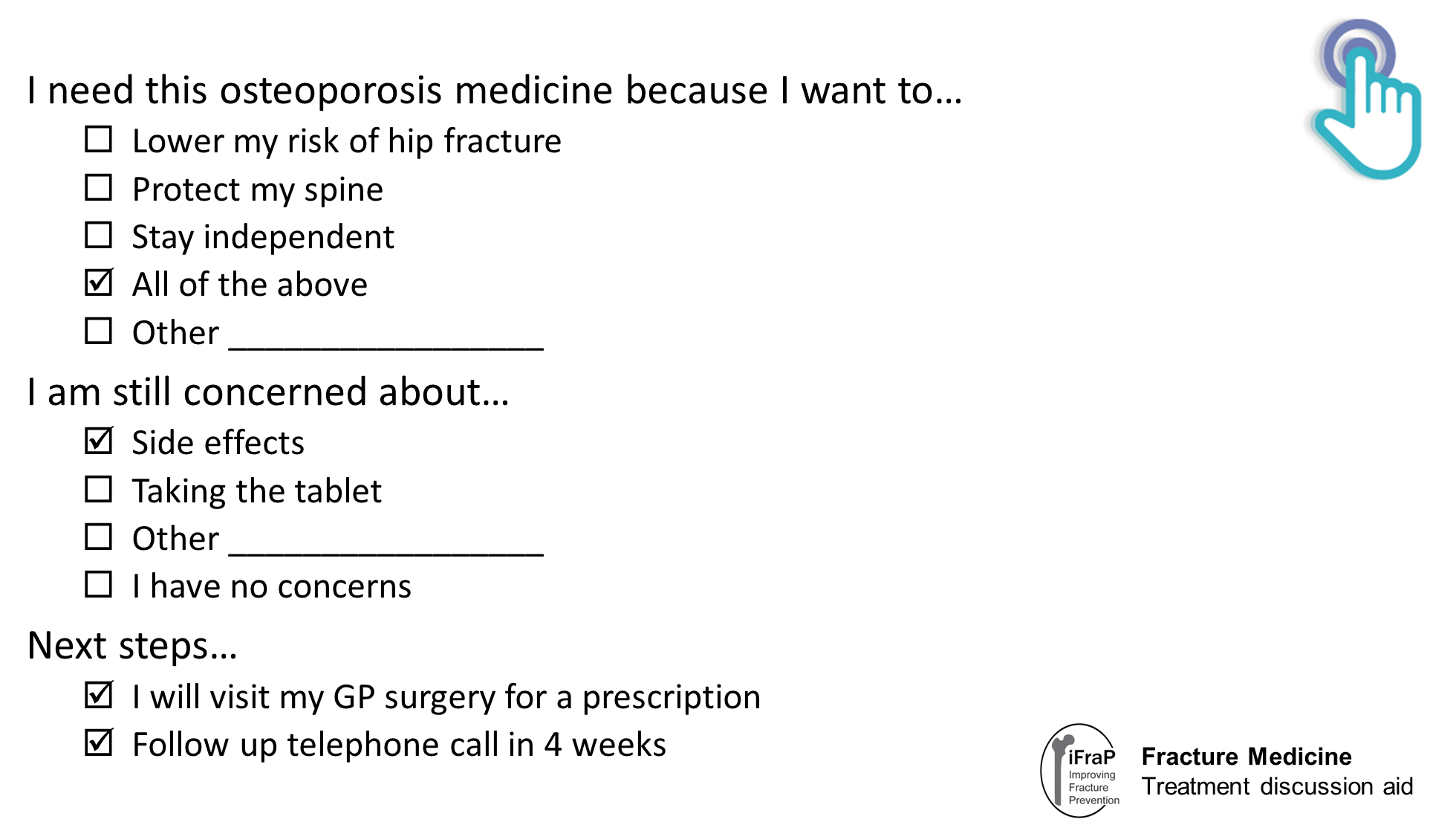


| **Additional file 3:** Analytical framework matrix mapped to the Theoretical Domains Framework domains, with example quotes | |
| --- | --- |
| **Inductive codes** | **Example Participant Quotes** |
| **Knowledge** | |
| Expertise of FLS | I think, without blowing our own trumpets, we're kind of like the experts really, aren't we? (FLS01) |
| Knowledge and understanding of osteoporosis | I think it might be useful if we then get a summary of what's been discussed [in FLS] (…) when we have the conversation then, we can say, 'We can see that you've discussed X, Y and Z.' It's clear what's been discussed already (GP06)  yea, I do wonder whether I know enough to explain it back to the patient (GP03)  The side effects outweigh the goodness that it does because I still don't know what it does (P03) |
| Explanations of osteoporosis | I would have said that she went through the questionnaire [of history taking] and again there was no explanation as to some of the questions, I put two and two together later (P05)  Would they have to swallow the tablet whole with a large glass of water and not have any food for 30 minutes? Saying that to a patient is fine but I think it's much better if you give an explanation as to why we're asking them to do that (FLS01) |
| **Interpersonal and cognitive skills** | |
| Clinical skills and training | It’s about actually developing a consultation skill that would involve the use of a tool like this. It’s going to change how we do an assessment or a consultation (FLS09) |
| Clinical skills and training  Explanations of osteoporosis | I recognise that it's something that we *could* do but currently we don't. I don't think we find out what their values are before or what their expectations are. (FLS01)  I've heard about osteoporosis, but I didn't know exactly what osteoarthritis was. (P03) |
| Explanations of osteoporosis  Personalised approach | You try and make it as basic as possible for some individuals and just keep the information that you give them as low key and as easy as possible. (FLS08) |
| Perception of risk | I think in some ways because it goes against the figures that you’re given for taking treatments a little bit. Most of them say it will reduce their risk of having a fracture by 50% but that’s just showing 10%. It’s then about which information you’re going on (FLS09)  Once you start talking in numbers you just see people say 'No you've lost me. That doesn't mean anything to me.' (GP07)  I’m quite happy with percentages. Yeah, I think probably percentages would be as good as anything to me (P08) |
| **Physical skills** | |
| Ability to use technology  Clinical skills and training | It looks pretty straightforward, doesn't it? We’re all pretty IT savvy, aren’t we? (FLS02)  After the last few weeks of Zoom and WhatsApp video I don’t think I’d mind. Prior to that I might have said that I preferred the telephone [laughs], but I don’t think I’d mind now (P08) |
| **Memory, attention, and decisional processes** | |
| Preparedness | I didn’t know what else to expect other than that, I don’t think I expected to be given a diagnosis there and then. (P05)  I didn't know what to expect really, so I was being guided by her. I didn't know that if you had bone fragility that you'd have to take something really big for it. I didn't ask that question because, again, it's only the first time it's happened to me and so I hadn't got a clue (P03) |
| Amount of information  Importance of visual information | It really depends on patients. You’re given quite a lot of information and you get to a point you consider that they’re a bit saturated (FLS08)  I think to have something visual like this would help to split the discussion up into groups so you are going to be able to remember it better and because it’s more categorised I think again that’s going to help to organise your thoughts which is better for retention, isn’t it? (P05) |
| Decision-making about medicine  Clinical skills and training | Do you treat them? Don’t you treat them? We will sit and debate it, won’t we? (FLS09)  We have an MDT with rheumatology and our neighbouring CNS in [hospital] and we all come up with a different point of view as to what is actually the best way to manage this individual. Sometimes, like you say, it’s a really, really grey area, isn’t it? (FLS08)  we normally just ask our consultants for their opinion before we will consider a recommendation and what recommendation is best and appropriate for this patient (FLS06) |
| Patient involvement in treatment decisions | I don’t know that I’ve ever come across a patient that’s come to me and said, ‘I want to start on this medication or that medication.’ I don’t know that I’ve had anybody that’s researched it that much (FLS08)  I would like to have had the discussion about the different sorts, yes. Just to feel more informed about the condition and ways of dealing with it (P05)  I suppose it [DST/icon array] might feel like they're being more involved in making the decision. 'These are the pictures. We don't know where you're going to sit. What do you think?' (GP07) |
| **Environmental context and resources** | |
| Connections, commonalities, and inconsistencies across services | It isn't very consistent, is it, if my experience is totally different from what you're getting at the [hospital]? Across the country, it must be very inconsistent. (GP03)  I think it [iFraP DST] would help with consistency (FLS01)  Obviously every service is different, isn't it? (FLS06) |
| Time constraints | Potentially, I think it would make the assessment a lot longer. I guess it’s something for if a patient came up with a specific question. (FLS03)  I think it’s going to make the consultation longer with this one. (FLS01) |
| Accountability of limited resources | We got this directive from pharmacy and NHS England that we've got to prescribe the cheapest one. There is not a lot of discussion really. This is the one we have to use. (GP3)  We will present first what is our first line of treatment. Obviously, we have to consider the cost. Normally, we do recommend alendronic acid (FLS01) |
| **Social influences** | |
| Social support and influence | You also get quite a few patients that have already come with preconceived ideas because their friend or neighbour has already been on that medication and they've already got ideas that they're not going to have it because it didn't suit such and such a person. (FLS01)  Although the person might be reluctant, quite often it will be the relative that says, ‘It’s only once a week.’ (FLS09)  I would have preferred either to have had somebody with me, possibly, who you know would have taken the information in. (P05) |
| **Social/professional role and identity** | |
| Patient involvement in treatment decisions | FLS01: Sorry, no, they don't have a choice [laughter]. FLS02: We say, 'This is what we'd recommend your GP prescribes for you. That's the treatment that would be recommended for you.' |
| Lifestyle as part of the treatment package  Clinician roles | You’re starting the treatment straightaway by educating them about their lifestyle, like not smoking, limiting their alcohol intake and exercise. I think that’s considered as a treatment already by giving them lifestyle advice. (FLS06)  I sometimes think services like the Fracture Liaison very much get a snapshot of the person because you have that short period of time focusing on one problem. 'Here's the problem. Here's the solution. This is what we do about it.' Perhaps, maybe when someone comes back to us, we put it more into context of what else is going on in their lives and see if it fits. (GP07) |
| **Beliefs about consequences** | |
| Patient involvement in the consultation | I get where you’re coming from and that the patients need to be at the centre of this assessment but for clinicians to gather the information that can form a management plan, there has to be a certain structure to that assessment. Otherwise, if we’re going to leave it to the patient to say what’s important to them, we may lose quite a lot of information that enables us to give them the best clinical pathway for them FLS08)  FLS09: I think you’d also tend to focus on the computer than the patient. FLS08: I agree. I think [FLS07] said it earlier, that one thing they don’t like about the GP is that the GP looks at a computer screen and doesn’t look at them. |
| Perceptions of risk | Only ten people are going to avoid breaking a bone and we’re starting them on this medication with all these side effects. If it was me, I would say, ‘Is it worth me taking it?’ (FLS08) |
| **Beliefs about capabilities** | |
| Decision-making about medicines  Trust in the clinician  Views of healthcare services | Yeah, probably for our first line of treatment, we generally tend to go for alendronic acid, If they've got no risk factors for that. If they say to us that they have mild acid reflux, we might refer for risedronate, an alternative bisphosphonate. (FLS01)  I daresay nowadays that a lot of the nurses, they have to take extra qualifications that they’re kind of almost coming, you know, practitioners in their own right, aren’t they? (P07)  The [Fracture Liaison] service… seems to be very good (…) It looks like we're spoilt [laughter]. (GP04) |
| Decision-making about medicines | In that situation, we normally just ask our consultants for their opinion before we will consider a recommendation and what recommendation is best and appropriate for this patient. We’ve got access to consultants every day and that’s really good. (FLS05) |
| Ability to use technology | My mother might have more difficulty doing it [using the DST]. I’m afraid this is one of my hobby horses at the moment, that we’re all being expected to be computer literate and many people over 80 aren’t and are feeling slightly excluded. (P08)  We’re very au fait with IT but... I’m just thinking of my mum who is hopeless with computer screens. She has no idea. (FLS09) |
| **Intentions** | |
| Perception of risk  Importance of visual information  Patient involvement in treatment decisions | I think it would generally help because people understand something pictorial much more than just figures. (FLS09)  I like the effectiveness button because that's something that's difficult to convey to patients and we've got nothing at the moment. I feel that would add something to our consultation (FLS01)  Obviously, you'd go through the effectiveness. You've got to go through that anyway. This might make it easier for you to show them (FLS03)  I suppose it [DST/icon array] might feel like they're being more involved in making the decision. 'These are the pictures. We don't know where you're going to sit. What do you think?' (GP07) |
| Expertise of FLS  Personalised approach | It would be good for newer staff, maybe, that don't know it all and they could click on there just as prompts but we kind of do it anyway (...) We're not gaining from it if it's just repeating something we do anyway in our FLS service and just clicking on and reading, that’s pointless, I suppose. If you're getting something extra like a video, then yeah (FLS04)  I don't think it's any different to what we already do (FLS02)  I wouldn't show it to every patient. I'd gauge who would probably be okay with it. You just know who would understand it. I wouldn't show it to everybody. (FLS03) |
| **Goals** | |
| Adherence to medicine | If they've got quite low bone density, to me, I want them on treatment. I want them to know and to recognise the importance really and so I will be quite direct and relay the fracture risk to them. (FLS03)  In the future, realistically with compliance, this could be emailed out to a patient and they just click, click, click and it would be amazing, wouldn’t it? (FLS08) |
| **Reinforcement** | |
| Connections, commonalities and inconsistencies across services | I suppose if there was a standardised leaflet and everyone who has been through FLS has had the same information and the information that we're giving them as GPs, because it will vary slightly between all of us (GP06)  The important thing is reinforcement, isn't it? We all don't take in everything we're told in one go (GP02)  If you knew which educational resource they'd had, it may guide you then when you speak to them. (GP01)  FLS01: Yes, I think it [iFraP DST] would help with consistency. FLS04: It's a way of measuring and everybody is doing the same thing… because we know we do it. I suppose a lot don't |
| **Emotion** | |
| Patient emotional reaction  Importance of medicines  Explanations about osteoporosis | ‘You really frightened me because I was convinced I was going to have another fracture.’ I think following that conversation with her, I try and suit it to each patient now (FLS07)  I think that percentage of fracture risk is really powerful for the patient to start treatment and to take it. If you’re saying, ‘You’ve got a 15% chance of fracturing your hip. Whereas your risk of side effects from the medication is lower,’ that’s powerful. (FLS09)  If they're worried about having osteoporosis, I say, 'It's a good thing that we've identified it. There are good treatments out there that can help prevent osteoporosis and improve bone density.' (FLS01) |
| Concerns about medicines | We have to say, ‘This is the risk of taking this treatment. This is what could possibly happen.’ As soon as they hear that, they’ll say, ‘Gosh! I’m not having that.’ If you say, ‘There is a side effect of acid indigestion,’ and they’ll say, ‘I’ve already got that, so I’m not having it any worse.’ (FLS07) |
| Unique identifiers for patients [P], Fracture Liaison Service (FLS) clinicians [FLS] and General Practitioners [GP]. | |

1. Paskins Z, Torres Roldan VD, Hawarden AW, Bullock L, Meritxell Urtecho S, Torres GF, Morera L, Espinoza Suarez NR, Worrall A, Blackburn S, Chapman S. Quality and effectiveness of osteoporosis treatment decision aids: a systematic review and environmental scan. Osteoporosis International. 2020 Oct;31(10):1837-51. [↑](#footnote-ref-1)
